# Heme oxygenase-1 in the forefront of a multi-molecular network that governs cell–cell contacts and filopodia-induced zippering in prostate cancer

**DOI:** 10.1038/cddis.2016.420

**Published:** 2016-12-29

**Authors:** Alejandra V Paez, Carla Pallavicini, Federico Schuster, Maria Pia Valacco, Jimena Giudice, Emiliano G Ortiz, Nicolás Anselmino, Estefania Labanca, Maria Binaghi, Marcelo Salierno, Marcelo A Martí, Javier H Cotignola, Anna Woloszynska-Read, Luciana Bruno, Valeria Levi, Nora Navone, Elba S Vazquez, Geraldine Gueron

**Affiliations:** 1 Department of Biological Chemistry, School of Sciences, FCEN, University of Buenos Aires, IQUIBICEN-CONICET, Buenos Aires, Argentina; 2 Department of Physics, FCEN, University of Buenos Aires, IFIBA-CONICET, Buenos Aires, Argentina; 3 Department of Cell Biology and Physiology, School of Medicine, University of North Carolina at Chapel Hill, Chapel Hill, NC, USA; 4 Department of Genitourinary Medical Oncology, The University of Texas, MD Anderson Cancer Center, Houston, TX, USA; 5 Pharmacology and Therapeutics Department, Roswell Park Cancer Institute, Buffalo, NY, USA

## Abstract

Prostate cancer (PCa) cells display abnormal expression of cytoskeletal proteins resulting in an augmented capacity to resist chemotherapy and colonize distant organs. We have previously shown that heme oxygenase 1 (HO-1) is implicated in cell morphology regulation in PCa. Here, through a multi 'omics' approach we define the HO-1 interactome in PCa, identifying HO-1 molecular partners associated with the integrity of the cellular cytoskeleton. The bioinformatics screening for these cytoskeletal-related partners reveal that they are highly misregulated in prostate adenocarcinoma compared with normal prostate tissue. Under HO-1 induction, PCa cells present reduced frequency in migration events, trajectory and cell velocity and, a significant higher proportion of filopodia-like protrusions favoring zippering among neighboring cells. Moreover forced expression of HO-1 was also capable of altering cell protrusions in transwell co-culture systems of PCa cells with MC3T3 cells (pre-osteoblastic cell line). Accordingly, these effects were reversed under siHO. Transcriptomics profiling evidenced significant modulation of key markers related to cell adhesion and cell–cell communication under HO-1 induction. The integration from our omics-based research provides a four molecular pathway foundation (ANXA2/HMGA1/POU3F1; NFRSF13/GSN; TMOD3/RAI14/VWF; and PLAT/PLAU) behind HO-1 regulation of tumor cytoskeletal cell compartments. The complementary proteomics and transcriptomics approaches presented here promise to move us closer to unravel the molecular framework underpinning HO-1 involvement in the modulation of cytoskeleton pathways, pushing toward a less aggressive phenotype in PCa.

Prostate cancer (PCa) is the most frequently diagnosed cancer in men aside from skin cancer.^1^ Although PCa has been well defined in regards to the mutational landscape, analysis at the proteome level of these genetic alterations is still understudied. Most of the functional information of the cancer-associated genes relies in the proteome, an exceptionally complex biological system involving several proteins that function through dynamic protein–protein interactions and post-translational modifications.^2^

Tumor development and progression are partly consequence of defects in mechanisms controlling cytoskeletal remodeling.^3^ Actin re-arrangement and attachment to focal adhesions at the leading edge of a migrating cell, generate the driving force necessary for movement.^3^ The loss of cell–cell adhesion enables cancer cells to dissociate from the primary tumor mass and changes in cell–matrix interaction allows the cells to invade the surrounding stroma.^4^ Higher grade prostate carcinoma has been associated with the loss of cell adhesion molecules at adherens junctions.^5^ Cell protrusive forces are partly regulated by the GTP-binding protein Rac.^6^ The delicate equilibrium between the cell pushing and pulling forces drive leading edge dynamics and cell migration. Interdigitating filopodia are vital for the proper alignment and establishment of the initial cell–cell adhesions^7^ This event is known as adhesion 'zippering'.^8^

Heme oxygenase 1 (HO-1) is the rate-limiting enzyme in heme degradation.^9, 10^ HO-1 is as a stress response protein and a critical mediator of cellular homeostasis.^11^ Although the role of HO-1 in cancer is controversial,^12^ we have shown that its pharmacologic or genetic upregulation is associated with a less aggressive phenotype in PCa.^13^ HO-1 inhibits cell proliferation, migration and invasion,^14^ it impairs tumor growth and angiogenesis *in vivo* and downregulates the expression of target genes associated with inflammation.^14, 15^ HO-1 is also implicated in the modulation of cellular adhesion in PCa, upregulating E-cadherin and *β*-catenin expression, and relocating them to the cell membrane,^13^ favoring a more epithelial phenotype. However, it is yet unclear which are the HO-1 interactors and how it regulates cytoskeleton organization.

In this work, we undertook a proteomics study to build the HO-1 interactome in PCa. We showed that HO-1 binds to key factors associated with the dynamics of the actin cytoskeleton and induces the remodeling of the actin filament architecture at filopodia. Further we obtained RNA-sequencing (RNA-Seq) profiles and determined a significant alteration of cytoskeletal genes by HO-1 induction, establishing molecular pathways by which forced expression of HO-1 leads to a more adhesive and less invasive phenotype, further supporting the antitumoral function in PCa.

## Results

### Proteomics profiling of HO-1-interacting proteins in PCa cells

**Figure 1:**
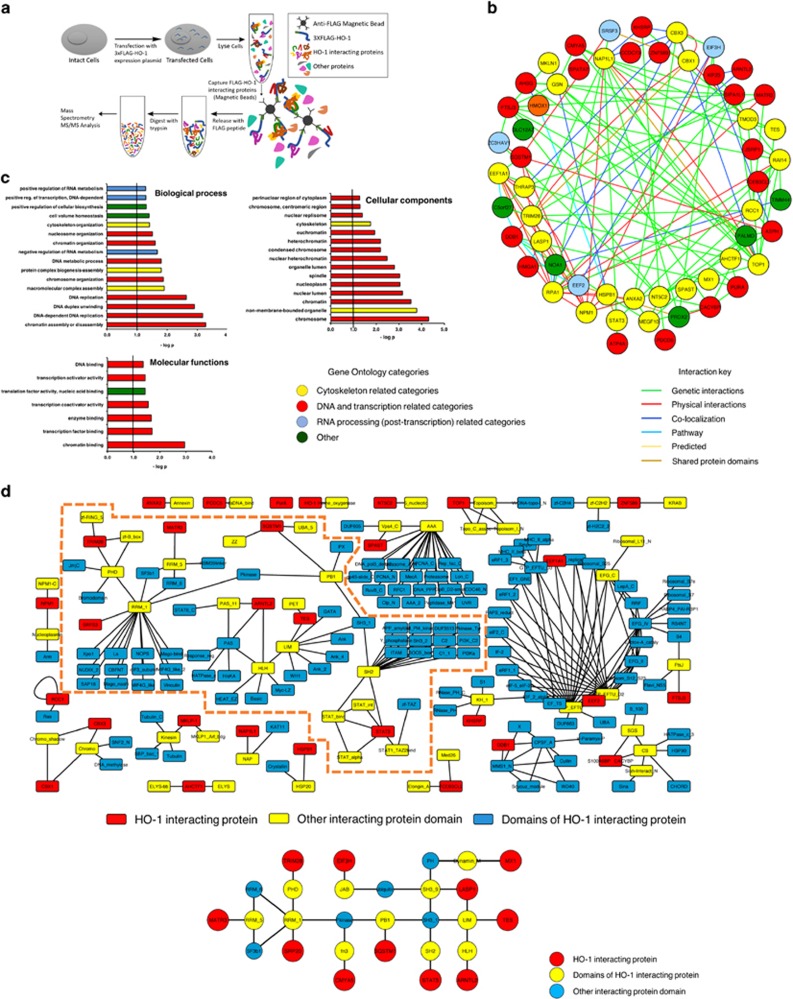
Construction of the HO-1 interactome in PCa cells. (**a**) Simplified schematic workflow of the construction of the HO-1 interactome in PC3 cells. FLAGHO-1 immunoprecipitation assays were performed from PC3 cell extracts that had been previously transiently transfected with FLAGHO-1 plasmid or empty FLAG vector and treated with H_2_O_2_ (200 *μ*M, 1 h). For LC-ESI MS/MS analyses, peptides were desalted and concentrated using a C18 resin. Peptides were analyzed by reverse-phase chromatography before mass spectrometry analysis. The peak lists obtained were processed and analyzed with NCBI databases using Mascot Software (Matrix Science Inc, Boston, MA, USA) and compared with the human genome, with a fragment ion mass tolerance of 0.80 Da and a parent ion tolerance of 2.0 Da. (**b**) Protein interaction network of HO-1 interactome in PC3 cells displaying interconnectivity either by reported gene colocalization, genetic interactions, predicted functional relationship, shared protein domains or physical interaction (left panel). Protein interaction network shows physical interconnectivity between HO-1-interacting proteins (right panel). (**c**) GO analysis of HO-1-interacting proteins was performed using DAVID software (https://david.ncifcrf.gov/). The resulting categories for biological process, cellular components and molecular functions were further sub-clustered into four global categories named: 'DNA and chromatin' (red), 'RNA processing' (blue), 'actin and cytoskeleton' (yellow) and 'other function' (green). (**d**) Schematic representation of the inferred domain–domain interactions of HO-1-interacting proteins. Reference amino-acid sequences of protein domains of HO-1 interactors were aligned against the Protein Data Bank (PDB). The crystallographies with the highest coverage and score were selected and introduced into the protein domain database (PFAM). HO-1 candidate interactors protein domains were annotated. Individual searches in the Interaction Protein Family (iPfam) database were performed. The networks were built using Cytoscape 3.2.1 software showing 201 nodes (rectangles) and 327 edges (black lines). The interactome network (framed in orange dashed-line, upper panel) was simplified and consists of 28 nodes (circles) and 29 edges (black lines) (bottom panel) representing: yellow circles, domains of HO-1-interacting proteins; red circles, HO-1-interacting proteins; light blue circles, other protein domains interacting with domains of HO-1-interacting proteins

We have previously demonstrated HO-1 implications on the maintenance of the epithelial cell morphology and architecture.^13^ We hypothesized that HO-1 could be exerting a regulatory role through its interaction with cytoskeleton-associated proteins. For this purpose, we conducted an in-depth mass spectrometry-based proteomics study. We constructed a recombinant FLAGHO-1 protein. PC3 cells were transiently transfected with FLAGHO-1 or the respective controls and treated with H_2_O_2_. Immunoprecipitated protein complexes were subjected to LC-ESI MS/MS (Figure 1a). This approach rendered a list of 56 proteins (Table 1). We performed a protein interaction network, showing interconnectivity either by reported gene colocalization, genetic interactions, predicted functional relationship, shared protein domains or physical interaction (Figure 1b, right panel). In all, 53% of the HO-1 interactome showed physical interconnectivity. The gene ontology (GO) analysis revealed an enrichment of proteins associated with four main functions: DNA and chromatin, RNA metabolism (post-transcription, including splicing), actin and cytoskeleton proteins and other proteins (Figure 1b, left panel).

### Crystallographic analysis of HO-1-interacting proteins

To assess protein domain interaction of HO-1 interactors, we obtained their reference amino-acid sequences. The crystallographies with the highest coverage and score were selected and introduced into the protein domain database (PFAM) (Supplementary Figure S1). Using the inferred domain–domain interactions, we restricted the analysis to those proteins presenting domain interconnectivity, obtaining a network with 28 nodes and 29 edges (Figure 1d). We could not predict domain interaction between HO-1 and its interactome because HO-1 is a small globular protein with only one reported domain. However, we successfully predicted domain interactions among several HO-1-interacting proteins: LASP1 and TES through the LIM domain, TES and ARNTL2 through LIM and HLH domains and TRIM28 and SRP20 through PHD and RMM_1, (Figure 1d). The crystallographic analysis of protein domains further supports physical interconnectivity of HO-1 interactor proteins.

### Analysis of multiple microarray data sets for cytoskeletal HO-1-interacting proteins

**Figure 2:**
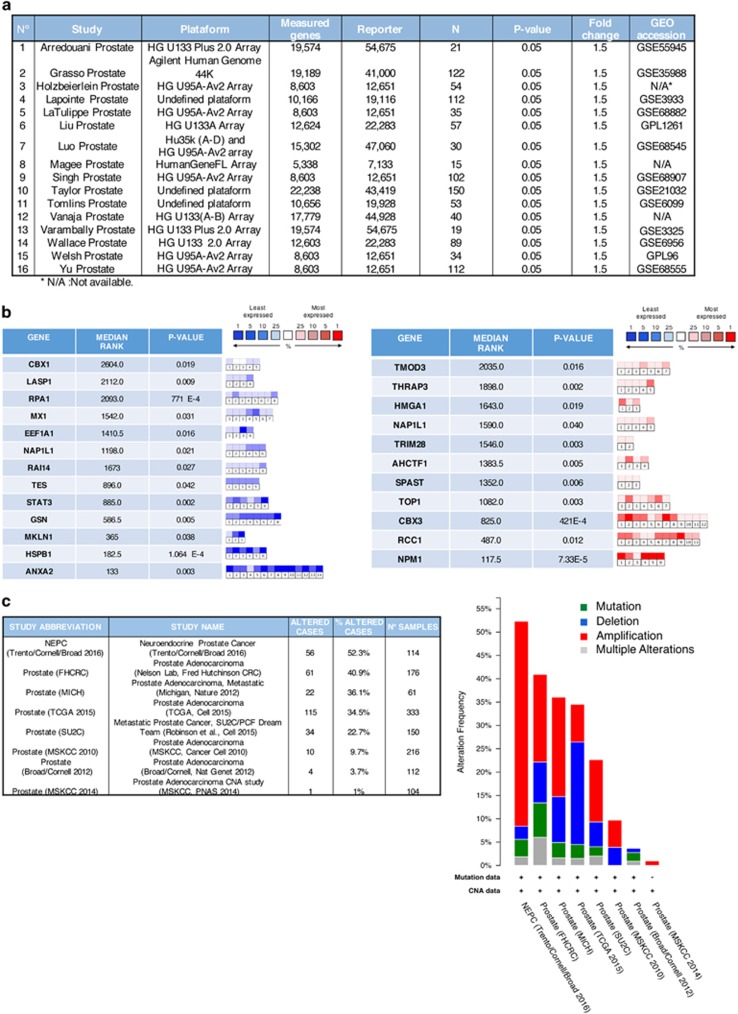
Meta-analysis of multiple microarray data sets for cytoskeletal HO-1-interacting proteins. (**a**) Expression microarray studies selected from the Oncomine platform (http://www.oncomine.org) comparing prostate adenocarcinoma *versus* normal prostate. (**b**) Summary table showing the gene name, median gene rank and corresponding *P*-value for the HO-1 cytoskeleton-related interacting proteins. The median rank for a gene is the rank for the gene across each of the analyses. The *P*-value for a gene is the *P*-value for the median ranked analysis. Right panel shows a heat map indicating the level of expression for each gene in each study selected (blue: least expressed, red: most expressed). Each square represents the number of studies that met our eligibility criteria and thresholds. Colors are z-score normalized to depict relative values within a row. (**c**) Studies selected from the *cBioPortal for Cancer Genomics* platform (www.cbioportal.org) summarizing the total number of cases with alterations for each study (left panel). The graph (right panel) shows the percentage of altered cases (*y* axis) and the alteration type observed whether it corresponds to mutations or copy number alterations (CNA) for each study (*x* axis). Mutations are shown in green, deletions in blue, amplifications in red and multiple alterations in gray, for the cytoskeleton-related proteins mentioned in (**b**) across the seven data sets selected comparing prostate tumor tissue *versus* normal prostate

Given that HO-1 modulates the cell adhesive properties and morphology,^13^ we focused on HO-1-interacting proteins associated with the cytoskeleton organization. To address their relevance in PCa, we searched the public cancer microarray database, Oncomine (http://www.oncomine.org) (Figure 2a). The expression profile for these genes showed significant dysregulation for most of the cytoskeletal interactors of HO-1, in particular, upregulation for TMOD3, TOP1, CBX3, RCC1, NPM1 and downregulation for ANXA2, HSPB1, STAT3 and TES (fold change ⩾1.5, *P*-value⩽0.05; Figure 2b). The meta-analysis combining data from the independent data sets showed that the above-mentioned dysregulated cytoskeletal genes for prostate adenocarcinoma *versus* normal prostate gland lie within the 25% of the most consistently high- or low-expressed genes across this comparison. Of note, TES and MKLN lie within the 1–9% of the lowest expressed genes in PCa *versus* normal tissue (Figure 2b).

We also used the *cBioPortal for Cancer Genomics* platform (www.cbioportal.org) to search for the most common genetic alterations for these cytoskeletal proteins in PCa (Figure 2c, left panel). Results show presence of mutations, amplifications and deletions for the cluster of cytoskeletal genes selected. Of note, amplification was the most frequent alteration across the data sets (Figure 2c, right panel).

### PCa cell 2-D migratory patterns under forced expression of HO-1

**Figure 3:**
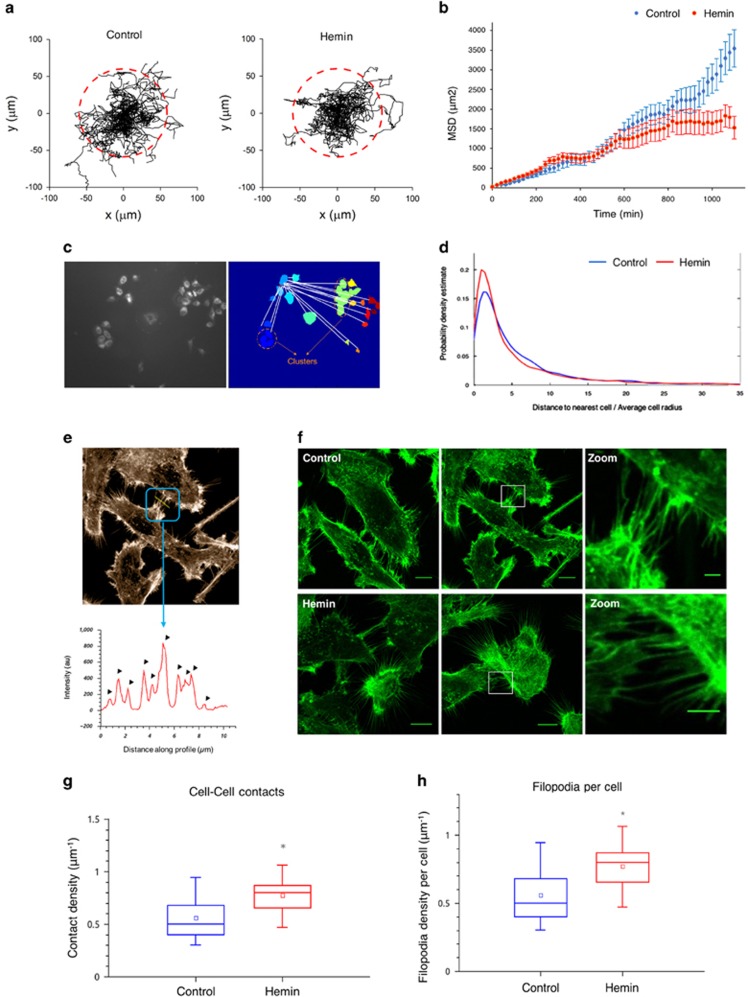
HO-1 overexpression impairs cellular trajectories in PCa cell lines and modulates the filopodia-like protrusions among neighboring PCa cells. Control and hemin (80*μ*M, 24 h) treated PC3 cells were grown to confluence into a monolayer in 35 mm Petri dishes. A linear scratch wound was done along the culture plate and cell movement was monitored at a rate of 1 frame/20 min. (**a**) Representative cell trajectory plots obtained for control (left panel) or hemin-treated PC3 cells (right panel). Red dashed circles represent the mean area explored by PC3 cells after 1100 min. (**b**) Trajectories were analyzed to obtain the MSD (mean square displacement) plot for control or hemin-treated cells. Data represent the average MSD±standard error over time, obtained from the analysis of 78 and 70 trajectories of control and hemin-treated cells, respectively. (**c**) PC3 cells treated with hemin (80 *μ*M, 24 h) or vehicle (control) were fixed and stained with C-Laurdan and images of large fields containing several cells were acquired using a 10x objective in an Olympus Wide Field Microscope (left panel). The images were analyzed with an algorithm designed to binnarize the image and assign a label to each set of connected pixels. In the right panel, each color represents a different cell or cell cluster (orange dotted circles). For every cell or cell cluster, the distances to all the other cells/cell clusters (white lines) and to the first neighboring cell (red line) were calculated. Distances were normalized to the average cell radii. (**d**) The distribution of the distances to the first neighbor cell was plotted for control and hemin-treated PC3 cells. The *x* axis displays the cell–cell distances (using a representative cell radius as scale) and the *y* axis represents the corresponding probability amplitude (*N*⩾2200 first neighbor distances for each condition). (**e**) PC3 cells treated with hemin or vehicle (control) were fixed and stained with rhodamine–phalloidin. Cells were imaged by confocal microscopy. The regions, in which cell filopodia contacted two neighboring cells, were divided into segments where the distance between the cells remained constant (blue frame). An intensity profile (yellow line) for each of these sectors was determined using a custom made algorithm to count contacts (Matlab). Arrow heads represent the intensity peaks considered as filopodia. (**f**) Two representative images for each group are shown (scale bars: 10 *μ*m). White open boxes represent the zoomed images (scale bars: 2 *μ*m). (**g**) Boxplot comparing the cell–cell contact density for the different experimental conditions. (**h**) The individual cell filopodia density was measured in hemin-treated and control cells (*N*⩾91 for each condition). *Significant difference, *P*<0.001

To examine whether the association of HO-1 with proteins implicated in the integrity of the actin cytoskeleton had an impact on PCa cellular migratory trajectories, we evaluated quantitatively the motion of cells in a wound-healing assay. Cells were treated with hemin (specific inducer of HO-1, 80 *μ*M, 24 h). A linear scratch was done along the culture plate and cells were imaged using phase contrast microscopy at 1 frame/20 min during 48 h. Single-cell trajectories (Figure 3a) were analyzed to obtain the mean squared displacement (MSD) *versus* time plot in each experimental condition. Figure 3b shows that the MSD obtained in both conditions increases as a function of time; however, after 10 h, the MSD obtained in hemin-treated cells was significantly smaller than in control cells. HO-1 induction significantly reduces the area explored by PCa cells in the assayed temporal window compared with control cells.

### PCa cellular protrusions under HO-1 induction

To analyze whether there were variations in cell–cell interactions, we determined the distribution of the distances to the first neighboring cell under HO-1 modulation (Figure 3c). For every cell or cell cluster, we calculated the distance to all the other cells/cell clusters (white line) and to the first neighboring cell (red line) (Figure 3c). Results show that HO-1 induction in PC3 cells significantly reduced the distances to the first neighboring cell (*P*<0.05; Figure 3d).

We used confocal microscopy to study the changes in the cytoskeletal organization, in particular the cell adhesion zippering through the actin stress fibers at the leading edge level of PCa cells. To quantify contacts among cells, we selected regions in which the filopodia from two neighboring cells touched each other, considered as 'contacts', and divided these regions into segments where the distance between the cells remained constant (Figures 3e and f). An intensity profile for each of these areas was analyzed with a custom made algorithm to count contacts (Figure 3e). A '*contact density'* was defined for each region as the ratio between the number of contacts and the length of the profile. Results showed an increased contact density under HO-1 induction, favoring adhesion zippering (Figure 3g; *P*<0.05). Filopodia-like protrusions (cell filopodia density) were also enhanced by hemin treatment (Figure 3h; *P*<0.05).

**Figure 4:**
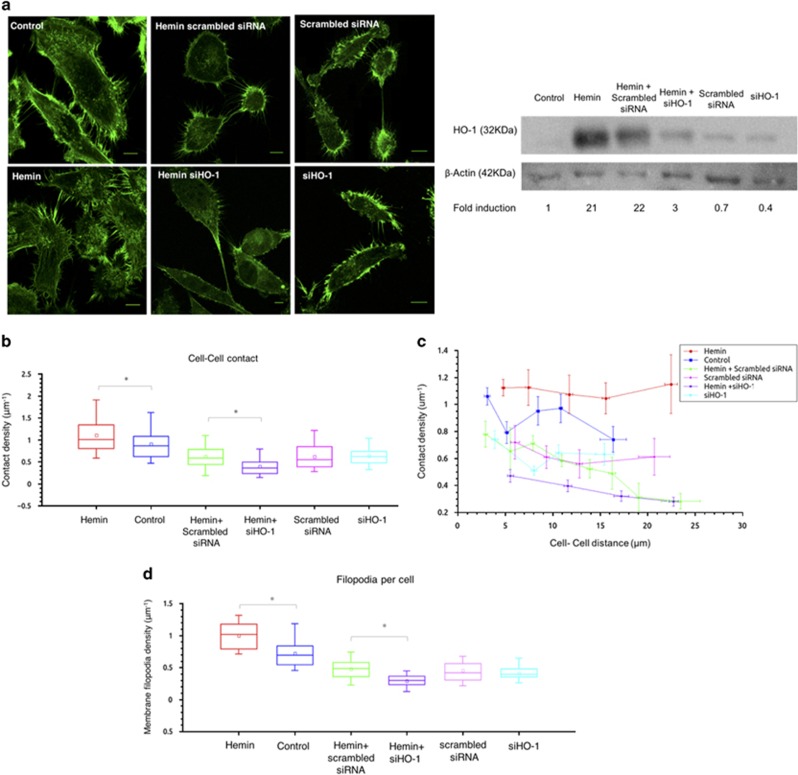
Filopodia-like protrusions under HO-1 modulation using siHO-1. (**a**) PC3 cells were treated with hemin (80 *μ*M, 24 h) or vehicle (control) and transfected with a specific siRNA for HO-1 (siHO-1) or scrambled siRNA (control). Cells were fixed and stained with rhodamine–phalloidin and imaged by confocal microscopy. One representative image for each group is shown (left panel). Efficiency of siHO-1 was confirmed by western blot. *β*-Actin was used as loading control (right panel). (**b**) Cell–cell contact density *versus* cell–cell distance. The amount of contacts between cells was obtained and a linear density was determined by computing the number of contacts per unit perpendicular distance. Each data point represents the mean contact density within a bin determined by Freedman–Diaconis rule and error bars represent standard error of the mean (*N*⩾33 contact regions for each condition). *Significant difference, *P*<0.001. (**c**) Boxplot comparing the cell–cell contact density *versus* cell distance for the different experimental conditions. The regions, in which cell filopodia contacted two neighboring cells, were divided into segments where the distance between the cells remained constant. An intensity profile for each of these sectors was determined using a custom-made algorithm to count contacts (Matlab) (*N*⩾33 contact regions for each condition; *P<0.001). (**d**) Boxplot comparing filopodia density on single cells for the different experimental conditions. The density was measured by scanning an intensity profile around the cell perimeter and evaluating the amount of filopodia per unit length (*N*⩾19 cells for each condition; *significant difference, *P*<0.001)

Using a specific siRNA for HO-1 (siHO-1), the effects observed on the augmented filopodia per cell and cell–cell contact, under HO-1 induction, were reversed (Figure 4a, left panel). Efficiency of HO-1 depletion was confirmed by western blot (Figure 4a, right panel). Accordingly cell zippering under HO-1 induction, was also impaired (Figure 4b). Contacts were further analyzed for the different distances among cells (range 1–25 *μ*m), displaying no negative correlation between cell–cell contacts and distance between cells for all treatments (Figure 4c). Moreover, HO-1 silencing also reverted the augmented filopodia density after hemin exposure (Figure 4d).

Presence of HO-1 at the leading edge protrusions of cells was also assayed by immunofluorescence. HO-1 induction in PC3 showed increased expression of this protein in the cytoplasm and nuclei of cells but did not reveal localization at cell filopodia (Supplementary Figure S2).

Metastatic cells rely on invadopodia to degrade and invade ECM.^5^ To rule out that these increased protrusions were invadopodia structures, we stained cells for metalloproteinase 9 (MMP9), a metalloproteinase highly implicated in tumor invasion. No staining for MMP9 was detected at these protrusions (Supplementary Figure S3).

Microtubule mechanics in PC3 cells treated or not with hemin was also evaluated by confocal microscopy (Supplementary Figure S4). The persistence length (pL) of microtubules was measured in PC3 cells. A Fourier analysis was performed and the ensemble variance of Fourier amplitudes was calculated. Both, cells treated or not with hemin, exhibited a similar thermal-like q-dependence: (1/Lp*)*(1/q)^2^ with no variation of Lp (Supplementary Figure S4).

Considering that PC3 cells present an osteolytic bone metastatic behavior, we extended our observations to C4-2B cells, another PCa cell line with osteoblastic metastatic behavior. Imaging of C4-2B cells also revealed an increased network of filopodia from neighboring cells and an augmented number of protrusions per cell by forced expression of HO-1 (Supplementary Figure S5). HO-1 depletion could revert the increased contact density under hemin exposure (Supplementary Figure S5), however, no significant difference was observed in the filopodia density, probably due to a reduced efficiency of the siHO compared with the effect observed in PC3 cells. These results show that HO-1 induction in PCa cells favors a more adhesive phenotype regardless the bone metastatic behavior.

### Analysis of PCa cellular protrusions in the presence of CM from co-culture systems (PC3 and MC3T3)

As bone is the most common and frequent site of PCa progression and the bioinformatics analysis has shown important dysregulation of cytoskeletal proteins in the metastatic stage of PCa, we next assessed the effect of conditioned media (CM) obtained from transwell co-culture systems (PC3 and MC3T3), on tumoral cell protrusions. MC3T3 is an osteoblast precursor cell line derived from mouse calvaria.^16^

**Figure 5:**
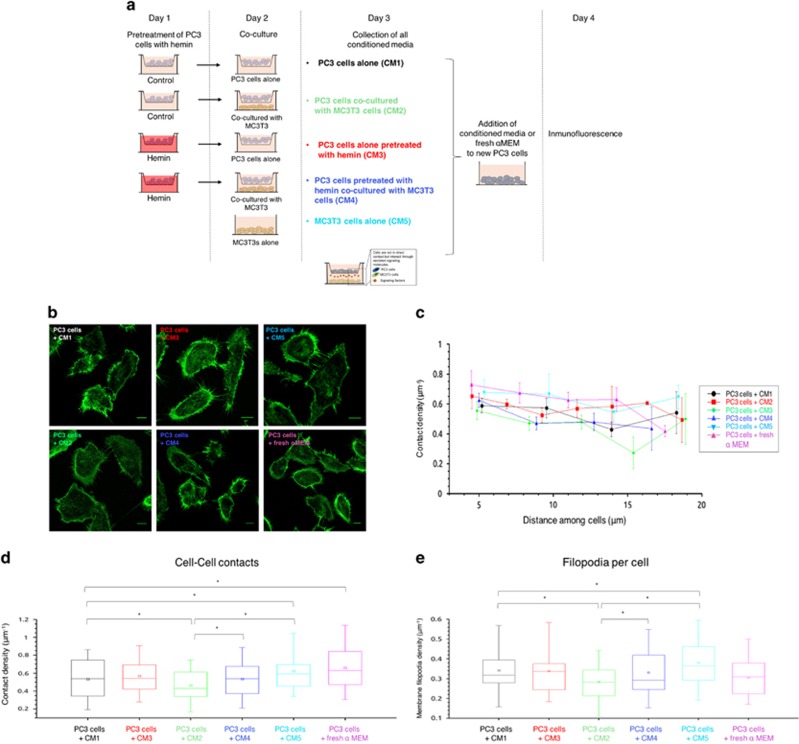
Analysis of cell contact density in PC3 cells cultured with CM from PC3-MC3T3 co-culture systems. PC3 cells were treated or not with hemin (80 *μ*M, 24 h) and then co-cultured with or without MC3T3 cells (24 h), in different compartments (insert membrane and well). CM from the different experimental conditions was then added to PC3 cells for 24 h. (**a**) Schematic representation of the experimental design. (**b**) Cells were fixed and stained with rhodamine–phalloidin and imaged by confocal microscopy to assess contact density and number of protrusions (scale bars: 10 *μ*m). One representative image for each group is shown. (**c**) Cell–Cell contact density *versus* cell–cell distance for all treatments. For each area where two cells were in contact, the amount of contacts was obtained and a linear density was determined by computing the number of contacts per unit perpendicular distance. Each data point represents the mean contact density within a bin determined by Freedman–Diaconis rule and error bars are the standard error of the mean (*N*⩾37 contact regions per condition). (**d**) Boxplot comparing cell–cell contacts densities for the different experimental conditions. An intensity profile for each of these sectors was determined using a custom made algorithm to count contacts (Matlab) (*N*⩾37 contact regions per condition; *P<0.001). (**e**) Boxplot comparing the measured filopodia density per cell for the different experimental conditions. Error bars represent standard error (*N*⩾36 cells per condition; *P<0.001)

PC3 cells pre-treated or not with hemin were co-cultured with or without MC3T3 cells. CM from the different experimental protocols was then added to PC3 cells (Figure 5a). Cell contact density and number of protrusions were evaluated by confocal microscopy (Figure 5b). Regarding contacts between cells, no correlation was observed for contact density and cell–cell distance for all conditions (Figure 5c). CM from co-culture systems reduced the number of contacts among PCa cells (Figure 5d; PC3+CM2 *versus* PC3+CM1) and hemin pre-treatment prevented this fall (Figure 5d; PC3+CM4 *versus* PC3+CM2). Intriguingly, the CM from co-culture systems impacted negatively on the membrane filopodia density of PC3 cells (Figure 5d; PC3+CM2 *versus* PC3+CM1). These effects were prevented by hemin pre-treatment (Figure 5d; PC3+CM4 *versus* PC3+CM2). These findings suggest that induced HO-1 expression in PC3 cells alters the soluble factors released to the CM in the co-culture systems, in turn affecting cell filopodia and zippering. A more adhesive phenotype of tumoral cells potentially prevent them from extravasation and invasion of other homing organs.

### Analysis of RNAseq data on PCa cells overexpressing HO-1 pharmacologically or genetically

**Figure 6:**
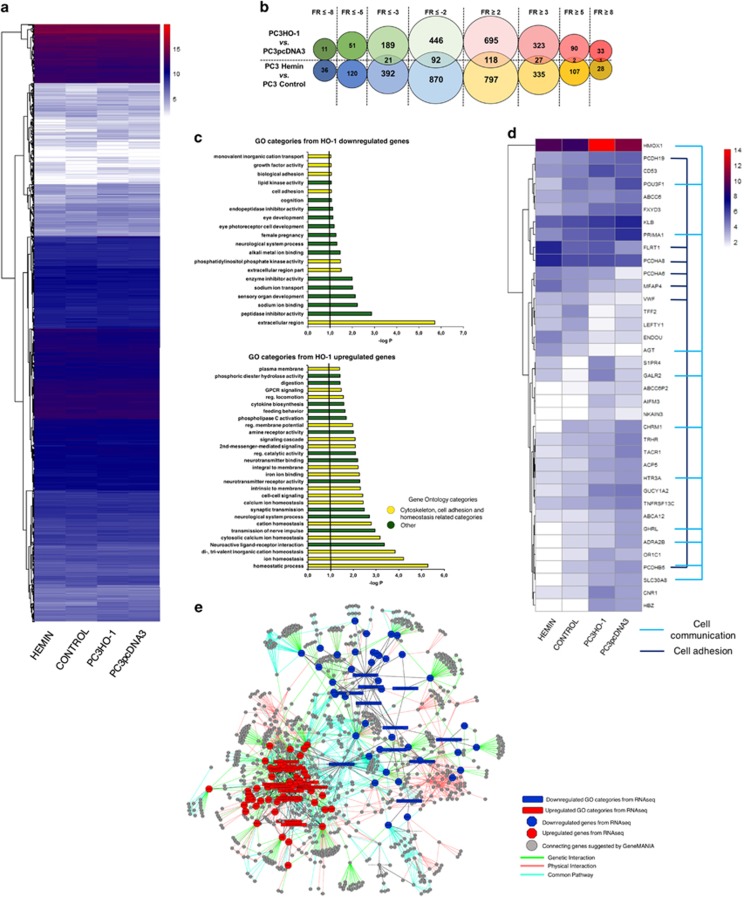
Analysis of RNAseq data of PCa cells overexpressing HO-1 pharmacologically or genetically. (**a**) RNAseq heat map of PC3 cells overexpressing HO-1 pharmacologically (Hemin) or genetically (PC3HO-1) and their respective controls. The transcripts were clustered in a conventional heat map analysis with red, blue and white plots representing transcripts with greater, intermediate and lower number of counts, respectively. (**b**) Schematic representation indicating the number and subset of upregulated (⩾2, ⩾3, ⩾5, ⩾8 PC3HO-1-red circles; PC3 hemin-yellow circles) and downregulated genes (⩽−2, ⩽−3, ⩽−5, ⩽−8, PC3HO-1-green circles; PC3 hemin-blue circles). The circle intersections show the number of overlapping genes between both conditions. (**c**) Functional analyses of differentially expressed genes by DAVID (https://david.ncifcrf.gov/). GO analysis on twofold-regulated overlapping genes between both conditions. The resulting categories were further sub-clustered into two global categories named: 'cytoskeletal, cell adhesion and homeostasis categories' (yellow) and 'other function' (green). (**d**) Heat map for the twofold overlapping differentially expressed genes for cytoskeletal-associated GO categories and other pathways relevant to cell adhesion. Red, blue and white plots represent transcripts with greater, intermediate and lower number of counts, respectively. Genes associated specifically with cell adhesion and cell–cell communication are marked in blue and light blue respectively. (**e**) Gene interaction network between the twofold overlapping HO-1 upregulated (red) and downregulated (blue) GO categories. Interconnectivity is depicted either by genetic interactions (green lines), physical interactions (red lines) or common pathways (turquoise lines)

Given the sound cellular evidence of HO-1 modulation on PCa cell adhesiveness and protrusive forces, we carried out an RNA-Seq analysis to compare gene expression profiles between PCa cells overexpressing HO-1 pharmacologically (hemin treatment) or genetically (transfected with pcDNA3HO-1) and their respective controls (Figure 6a). For both comparisons: PC3HO-1 *versus* PC3pcDNA3 (empty vector) and PC3 hemin *versus* PC3 control we obtained differential subsets of upregulated (⩾2, ⩾3, ⩾5, ⩾8-fold change cut-off) and downregulated (⩽−2, ⩽−3, ⩽−5, ⩽−8-fold change cut-off) genes (Figure 6b). We screened for overlapping up or downregulated genes for both comparisons (on same threshold subsets). Results show 92 downregulated (⩽−2, *P*<0.05) and 118 upregulated (⩾2, *P*<0.05) overlapping genes (Figure 6b). We selected these overlapping gene data sets and performed GO analysis identifying pathways in which these differentially expressed genes are involved and enriched in subcategories (Figure 6c). We performed a second heat map depicting the top 37 significantly regulated genes within these cytoskeletal-related categories (Figure 6d), highlighting key markers related to cell adhesion (blue) and cell–cell communication (light blue). We also built a transcriptomic interaction network, between the GO overlapping twofold upregulated gene categories (red) and twofold downregulated gene categories (blue) showing interconnectivity either by reported genetic interactions, common pathways, or physical interactions (Figure 6e).

### Integrated analysis of HO-1 transcriptomic and proteomics data in PCa

**Figure 7:**
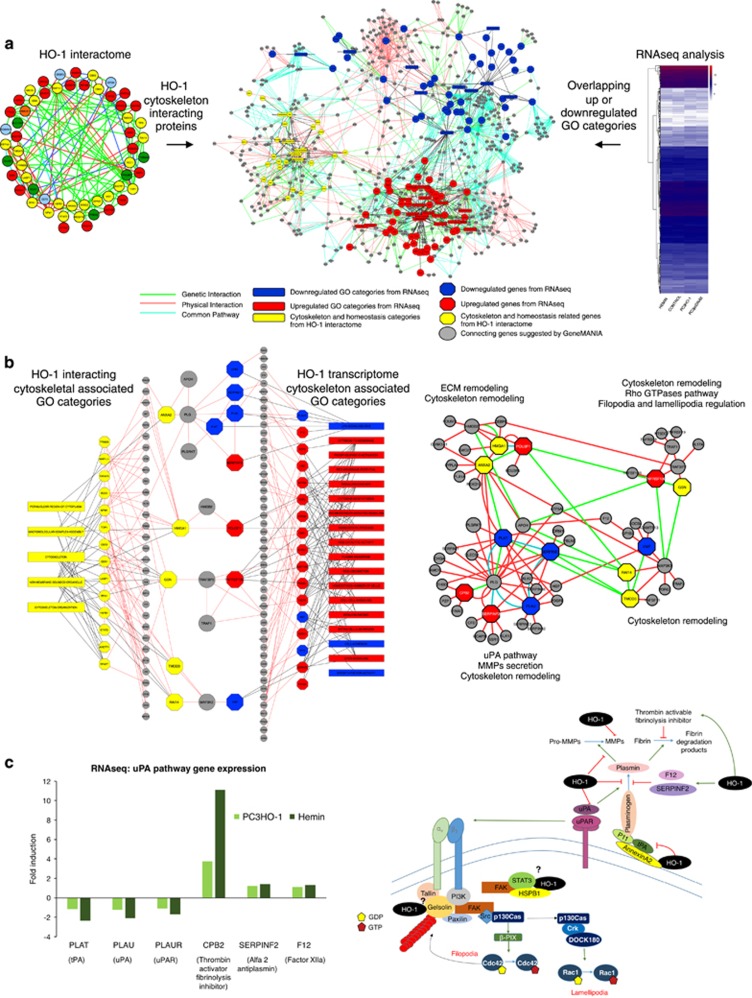
Integrated analysis of HO-1 transcriptomic and proteomics data in PCa. (**a**) Interaction network between proteins from cytoskeletal-related GO categories of the HO-1 interactome (yellow) and the twofold overlapping HO-1 upregulated (red) and downregulated (blue) GO categories from the RNAseq analysis. Interconnectivity is depicted either by genetic interactions (green lines), physical interactions (red lines) or common pathways (turquoise lines). (**b**) The above-mentioned interaction network was filtered by physical interconnectivity between proteins from cytoskeletal-related GO categories of the HO-1 interactome (yellow) and overlapping genes from the twofold upregulated (red) or downregulated (blue) cytoskeleton and homesotasis GO categories from the RNAseq analyses (left panel). Four nodes of connectivity between HO-1 interactome proteins and RNAseq data are displayed at the center of the figure (left panel) and expanded with there associated molecular and cellular pathways (right panel). (**c**) RNAseq expression profile for the urokinase-type plasminogen activator (uPA) pathway genes under HO-1 genetic (PC3HO-1- light green bars) or pharmacological (hemin – dark green bars) modulation (left panel). Schematic representation of the proposed model for the molecular pathways affected by HO-1 (right panel), resulting in impaired cell migration and invasion favoring a more adhesive and less aggressive phenotype in PCa. HO-1 overexpression impairs ECM degradation by downregulating MMPs, upregulating thrombin activable fibrinolysis inhibitor (TAFI), impairing fibrin degradation products. HO-1 also upregulates Alpha 2- anti-plasmin (SERPINF2), a serine protease inhibitor, responsible for inactivating plasmin, and downregulates uPA and urokinase receptor (uPAR). Downregulation of uPA/uPAR directly impacts on Rho GTPases pathways through the alpha V-beta 3 integrin receptor, which in turn affect lamellipodia and filopodia formation. HO-1 binds Gelsolin, STAT3 and HSPB1, potentially supporting its implication in lamellipodia and filopodia regulation

We next integrated the transcriptomic overlapping data sets with the HO-1 interactome for a combined holistic view of HO-1 molecular mechanisms implicated in the cytoskeletal remodeling in PCa. Figure 7a shows the interaction network between the cytoskeletal GO categories of the proteome with the overlapping twofold upregulated gene categories (red) and twofold downregulated gene categories (blue) of the trascriptome.

Filtering by physical interconnectivity among these data sets, four main molecular pathways were depicted: ANXA2 (Annexin 2) connected to PLAU (urokinase-type plasminogen activator precursor) signaling axis, HMGA1 (high mobility group AT-Hook) connecting to POU3F1, (POU class 3 homeobox 1) GSN (Gelsolin) with TNFRSF13C (tumor necrosis factor receptor superfamily member 13C) and TMOD3 (tropomodulin 3) and RAI1 (retinoic acid induced 1) with VWF (Von Willebrand factor) (Figure 7b, left panel). These pathways are intrinsically related to the cytoskeleton remodeling, MMP secretion, uPA pathways, Rho GTPases pathway and filopodia and lamillopodia regulation (Figure 7b, right panel). This network creates a molecular framework by which HO-1 operates at the molecular level governing cell protrusive forces, migration, invasion and cell adhesiveness.

Interestingly, uPA/uPAR, through the activation of the plasminogen system, degrade ECM, and consequently drive tumor cell membrane protrusion and motility.^17^ The RNAseq profiling for both PCa cells overexpressing HO-1 pharmacologically or genetically showed a direct regulation of the critical factors in the uPA/uPAR cascade, such as the downregulation of the axis activators: uPA/uPAR (PLAU/PLAUR) and tPA (PLAT), and the upregulation of the axis inhibitors: CPB2 (thrombin activator of fibrinolysis inhibitor), SERPINF2 (alpha 2 anti-plasmin, and F12 (factor XIIA). Our data delineate a molecular axis by which HO-1 potentially shuts down the acquisition of an invasive tumor cell phenotype, crucial for cancer metastasis.

## Discussion

Different sets of genes, proteins and metabolites govern progression from a precursor lesion, to the localized disease and finally to the metastatic stage. Although several studies have profiled PCa tissues at the transcriptome level, less work has been done at the protein level that serve as the functional effectors of cancer progression.

Several studies have shown the anti-inflammatory effects of HO-1 on diseases ^18, 19, 20^ although the underlying mechanisms are still to be deciphered. Its role in cancer is still controversial.^12, 21, 22, 23, 24, 25^ In PCa, HO-1 impairs cell proliferation, migration invasion, and angiogenesis *in vitro* and *in vivo*.^14^ The confirmation of its association with the augmented adhesive capabilities of cells and the induction and relocation of E-cadherin and *β*-catenin to the cell membrane,^13^ led us to hypothesize its potential implications in cell–cell adhesion zippering and in the regulation of the actin dynamics at the leading edge of cells.

For this purpose, we built for the first time the HO-1 interactome in PCa cells, showcasing 56 molecular partners, including cytoskeletal proteins with roles in cell structure, physiological processes, cell signaling and regulation of the actin stress fiber dynamics. Pharmacological HO-1 induction altered migratory patterns and PCa cell trajectories toward a less motile phenotype.

Cell–cell adhesion is essential for the development, differentiation and maintenance of tissues. Epithelial cells require cell–cell adhesion zippering to promote barrier function.^8^ This barrier is critical in the maintenance of cellular homeostasis. Its loss provides a considerable advantage for carcinoma progression, as disruption of cell–cell interactions and detachment from the ECM are required for proliferation, aberrant signaling, epithelial-to-mesenchymal transition, invasion and metastasis.^7^ These events are facilitated by the actin cytoskeleton and tubulin at microtubules.^26^ Actin is the key component of several cellular structures, including filopodia, lamellipodia and stress fibers. Actin bundling contributes to epithelial cell polarity by preserving cell–cell junctions and microvilli.^26^ In the metastatic disorder, actin and the cytoskeletal proteins serve as mechanosensors between the cell and the microenvironment.^26^ There is no general rule whether actin bundling promotes or inhibits cancer metastasis.^26^ Mechanical stiffness has long been positively associated with invasion and metastasis.^27^ However, Swaminathan *et al.*^28^ reported that cancer cells with the highest migratory and invasive potential are significantly less stiff than cells with the lowest migratory and invasive potential. It is clear that transformed cells display corrupt actin bundling, turning the cytoskeletal compartment into a key target to understand tumor progression and develop appropriate therapies.

By confocal microscopy, we quantified and compared actin filaments at the leading edge level in PCa cells. HO-1 induced the remodeling of the actin filament architecture at filopodia, yielding a more adhesive and less invasive phenotype. The fact that HO-1 silencing could revert the augmented contact density observed under HO-1 forced expression, showcases a direct effect on cellular actin-based protrusions. However, HO-1 displays negative staining at cell filopodia, pointing to a regulatory role of this protein on the actin filaments, rather than a direct interaction between these proteins.

Men with PCa display characteristically osteoblastic bone metastases, which are the main cause of morbidity and mortality of the disease.^29^ A previous report has shown HO-1 potentiality in modifying the bone microenvironment and subsequent PCa metastases.^30^ To explore the contribution of HO-1 in the interaction between PCa cells and osteoblasts, we used co-culture systems (PC3 and MC3T3). PCa cells exposed to CM from these co-culture systems, displayed reduced membrane filopodia density and contact among cells, effects reverted by hemin pre-treatment. Retraction of cell protrusions could be a sign of rounding up of cells and may be one of the characteristic features of cell detachment. Our results suggest that HO-1 prevents PCa cells from extravasation and invasion to other homing organs.

To point out the significance of the cytoskeletal HO-1-interacting proteins in prostate carcinogenesis, we searched the public cancer microarray database Oncomine and cBioportal. Essential transcriptional features for lethal castration-resistant PCa were discovered using meta-analysis of transcriptomic data.^31, 32^ Interestingly, TES and MKLN lie within the 1–9% of the lowest expressed genes across this comparison. TES, a scaffold protein that participates in the reorganization of the actin cytoskeleton,^33^ is known for being a tumor suppressor in PCa.^34^ MKLN1, a nucleocytoplasmic mediator of cellular morphology and adhesiveness,^35^ exhibits similar sub-cellular dynamics to HO-1, relocating from the cell membrane toward the cell nuclei under hemin treatment.^13^

Multi 'omics' technologies reveal new aspects of the mechanistic strategy that HO-1 uses to alter protrusive forces and adhesiveness of tumor cells, which may not otherwise be deduced from a single-omics approach. The RNA profiling evidenced significant alterations of key markers related to cell adhesion and cell–cell communication under HO-1 induction. Strikingly, when integrating the transcriptome under HO-1 modulation with the HO-1 interactome, a framework of four main molecular pathways arose as the foundation for the regulation of the tumor cell protrusive forces: ANXA2/HMGA1/POU3F1; NFRSF13/GSN; TMOD3/RAI14/VWF; PLAT/PLAU. We provide a schematic representation of the different cytoskeletal-associated targets modulated by HO-1 overexpression, which is supported by the results presented here and our previous data.^13, 14^ Of note, downregulation of uPA/uPAR directly impacts on Rho GTPases pathways through the alpha V-Beta 3 integrin receptor, which in turn affect filopodia formation. HO-1 binds Gelsolin, STAT3 and HSPB1, potentially supporting its implication in filopodia regulation and gives ground to HO-1 involvement at the molecular level in the modulation of the cytoskeleton pathways.

## Materials and methods

### Cell culture, treatments, reagents and antibodies

PC3 cells were obtained from the American Type Culture Collection (Manassas, VA, USA) and were routinely cultured in RPMI 1640 (Invitrogen, Grand Island, NY, USA) supplemented with 10% fetal bovine serum (FBS). PC3 cells were transiently transfected with HO-1FLAG plasmid or FLAG empty vector.

Hemin was obtained from Sigma-Aldrich (Glasgow, UK). For treatments, cells were incubated 24 h in RPMI media containing 10% FBS and then were exposed to hemin (80 *μ*M, 24 h).

For H_2_O_2_ treatment, cells were exposed to H_2_O_2_ (200 *μ*M, 0.5 h).

Polyclonal and monoclonal anti-HO-1 antibodies were from Stressgen Biotechnologies Corp. (San Diego, CA, USA). Anti-*β*-actin antibody was purchased from Sigma-Aldrich (UK). The rhodamine–phalloidin was purchased from Life Technologies (Thermo Fisher Scientific Inc., Eugene, OR, USA). Anti-mouse and anti-rabbit secondary antibodies conjugated with HRP were from Amersham Ltd (Freiberg, Germany). Secondary antibodies conjugated with Alexa Fluor 488 or Alexa Fluor 555 were from Molecular Probes, Invitrogen (Grand Island, NY, USA).

### p3xFLAG-CMV-10-HO-1 cloning

p3xFLAG-CMV-10-HO-1 vector was generated by cloning the cDNA sequence encoding the human HO-1 (HMOX1) into the restriction sites *Eco*RI and *Bam*HI of the mammalian expression vector p3xFLAG-CMV-10 (Sigma-Aldrich, UK). This strategy results in the fusion of the Flag tag peptide at the C-terminus of the HMOX1. The sequences of the primers used were as follow: forward – 5′-GCAGAATTCAGAGCGTCCGCAACCC-3′ reverse – 5′- GCCGGATCCGCATTCACATGGCATAAAGC-3′.

### FLAG immunoprecipitation strategy

PC3 cells were transfected either with FLAGHO-1 plasmid or the empty vector as a control. Forty-eight  hours after transfection, proteins were extracted using a buffer with a low NaCl concentration (20 mM Tris, 150 mM NaCl, 5 mM MgCl_2_, 0.5% NP40, pH 7,5) not to disrupt the protein–protein interactions. Protein extracts were incubated with Flag Magnetic Beads (purchased from Sigma-Aldrich, UK) for 2 h at 4 °C. After removing the proteins that did not interact with the FLAG construction, the proteins complexes formed were incubated with 3XFLAG peptide (100 *μ*g/*μ*l) for 2 h at 4 °C. Flag peptide competes with the proteins complexes bound to the magnetic beads and, as a consequence, the FLAGHO-1-interacting proteins remain in the supernatant.

### Separation of peptides and mass spectrometry

Recombinant FLAGHO-1 protein complexes were reduced (200 mM DTT), alkylated (200 mM iodoacetamide) and digested with trypsin in-solution overnight, using an estimated 1 : 30 enzyme to substrate ratio. The peptides were desalted and concentrated in a C18 resin (Zip-Tips, Waters Technologies Corporation, Milford, MA, USA) before analysis by LC-ESI MSMS at the Center for Metabolomics and Mass Spectrometry (The Scripps Research Institute, La Jolla, CA, USA). Peptides were separated by reverse-phase chromatography before mass spectrometry analysis using the following method: nanoelectrospray capillary column tips were made in-house by using a P-100 laser puller (Sutter Instruments, Novato, CA, USA). The columns were packed with Zorbax SB-C_18_ stationary phase (Agilent Technologies, Santa Clara, CA, USA) purchased in bulk (5-mm particles, with a 15-cm length and a 75-mm inner diameter). The reverse-phase gradient separation was performed by using water and acetonitrile (0.1% formic acid) as the mobile phases. The gradient consisted of 5% acetonitrile for 10 min followed by a gradient to 8% acetonitrile for 5 min, 35% acetonitrile for 113 min, 55% acetonitrile for 12 min, 95% acetonitrile for 15 min and re-equilibrated with 5% acetonitrile for 15 min.

Data-dependent MS/MS data were obtained with an LTQ linear ion trap mass spectrometer using a home-built nanoelectrospray source at 2 kV at the tip. One MS spectrum was followed by 4 MS/MS scans on the most abundant ions after the application of a dynamic exclusion list. Tandem mass spectra were extracted by use of Xcalibur software (Thermo Scientific, Waltham, MA, USA). All MS/MS samples were analyzed by using Mascot (version 2.1.04; Matrix Science, London, UK) with *H. Sapiens* proteins contained in the NCBI protein database, assuming the digestion enzyme trypsin. Mascot was searched with a fragment ion mass tolerance of 0.80 Da and a parent ion tolerance of 2.0 Da; identification was done at the 95% confidence level with a calculated false-positive rate of <1% as determined by using a reversed concatenated protein database. Peptide identifications were accepted if they could be established at a >95.0% probability as specified by the *Peptide Prophet algorithm*. Protein identifications were accepted if they could be established at a >99.0% probability and contained at least two identified peptides as specified by the *Protein Prophet algorithm*.^36^ Proteins that contained similar peptides and could not be differentiated based on MS/MS analysis alone were grouped to satisfy the principles of parsimony. To control for nonspecific binding, we compared FLAGHO-1 co-purifying proteins with those immunoprecipitated in cells transfected with a FLAG empty vector. Only differential FLAGHO-1 binding proteins compared with FLAG-binding proteins were considered further.

### Bioinformatics data analysis

In all cases, the networks were performed using Cytoscape 3.2.1 software (Institute of Systems Biology, Seattle, USA). GO analysis was performed using the Database for Annotation, Visualization and Integrated Discovery (DAVID) v6.7. (Leidos Biomedical Research, Inc., Bethesda, MD, USA).

### Hemin pre-treatment of PCa cells and Co-culture system

An *in vitro* bicompartment culture system was used as a model of bone metastases from PCa as previously described slightly modified. Briefly cells were seeded (100 000 cells) in cell culture inserts (0.4-mm pore; Falcon/Becton Dickinson Labware, Franklin Lakes, NJ, USA) and on day 1 they were treated with hemin (50 *μ*M, Sigma-Aldrich, St. Louis, MO, USA), a potent inducer of HO-1 (PC3 Hem). Controls received fresh medium. The MC3T3 cells were also seeded on day 1 in tissue culture plates (100 000 cells per well). On day 2, the inserts containing the PC3 cells (pre-treated or not with hemin) were extensively washed with PBS. Then, the inserts were placed into tissue culture plates containing MC3T3 so that the two different cell types shared the culture medium but were not in physical contact. Co-culturing of PC3 cells with MC3T3 was performed with *α*-MEM plus 2% FBS for 24 h. As control, each cell type (PC3 cells and MC3T3 cells) was grown alone. On day 3, all CM were collected and used to incubate new PC3 cells for 24 h so as to perform immunofluorescence and filopodia analysis on day 4. Cultures were done in triplicate and each experiment was assayed three times.

### siRNA transfection

siRNA anti-HO-1 (siHO-1) was used to knock down HO-1 expression and siRNA scramble was used as a negative control (ON-TARGETplus siRNA reagents, both purchased at Dharmacon, Pittsburgh, Pennsylvania, USA). The HO-1 siRNA is a pull of four targeted siRNA (HO-1 siRNA cat# L-006372-00-05, scrambled siRNA cat# D-001810-01-05). In all, 1 × 10^6^ PC3 cells were grown in 12-well plates until 60% confluence and then transfected using Dharmacon (US) transfection reagent (Dharmacon) in medium supplemented with 10% FBS without antibodies. One day after transfection, the medium was replaced for complete medium including or not hemin (80 *μ*M, 24 h). Forty-eight hours after transfection, cells were fixed and stained for immunofluorescence experiments.

### Bioinformatics inference on protein–protein interactions

Reference amino-acid sequences for EEF1A1, GSN, HSPB1, LASP1, MEGF10, MKLN1, MX1, NT5C2, RAI14, TES, TMOD3 (NP_001393.1, NP_001121135.2, NP_001531.1, NP_006139.1, NP_115822.1, NP_037387.2, NP_001171517.1, NP_001127845.1, NP_001138993.1, NP_056456.1, NP_055362.1, respectively) were annotated from RefSeq and a search for similar protein structures was achieved using Basic Alignment and Search Tool (BLAST) against the Protein Data Bank (PDB). The resulting structures were selected by assessment of the alignment coverage and score. Domain data were collected from the Protein Family database (PFAM). HO-1 interactor candidate protein domains were annotated and searches in the Interaction Protein Family (iPfam) database were performed, in order to get structural description of domain–domain interactions. The information gathered from these databases was used as input for the construction of an interaction network using Cytoscape 3.2.1 software.

### Bioinformatics analysis

#### Information source and eligibility criteria (Oncomine)

We searched the public cancer microarray database, Oncomine (715 data sets investigating and 68 tumor types), to identify expression microarray data sets that compared the expression of prostate adenocarcinoma *versus* prostate gland. In order to be included in our study, a data set was required to (1) be generated from human tumors, (2) compare prostate adenocarcinoma *versus* prostate gland, (3) have a *P*-value <0.05 and (4) have a fold change >1.5 and/or (5) have a gene rank between 1 and 10%. It is worth mentioning that although the *P*-value criteria was strict for the data set selection, some genes were considered even if the fold change or the gene rank was <1.5% or >10%, respectively, when the gene showed a significant over or under expression.

#### Search/study selection (Oncomine)

We performed one search for each gene using the 'gene symbol' as the search term and obtained different number of studies for each gene. The selected studies were analyzed on the basis of normal gland *versus* prostate adenocarcinoma. Cited literature was reviewed to confirm that the analysis was as documented in the Oncomine database.

#### Data source and selection criteria (CBioPortal)

The cBio Cancer Genomics Portal (http:/cbioportal.org), (open source cancer genomics data platform created by Memorial Sloan-Kettering Cancer Center (MSKCC)) was used to analyze the most common mutations of the selected genes in PCa. The criteria used in order to include data sets in our analysis were the following: (1) type of cancer: PCa; (2) the study must be published and (3) the study must consist of >60 samples.

#### Immunofluorescence experiments and quantitative microscopy

PC3 cells were fixed with 8% paraformaldehyde (PFA) (20 min, room temperature) and stained with rhodamine–phalloidin (1 h, room temperature). Confocal images were acquired by confocal microscopy (FV1000, Olympus, Tokyo, Japan) using an UPlanSApo 60x oil immersion objective (NA 1/41.35; Olympus), a diode laser of 543 nm as the excitation source and fluorescence was collected in the range of 555–655 nm. We selected the regions from which filopodia from two neighboring cells were in contact, considered as 'contacts', and divided these regions into segments where the distance between the cells remained constant. An intensity profile for each of these areas was analyzed with a custom made Matlab algorithm to count contacts. A 'contact density' was defined for each region as the ratio between the number of contacts and the length of the profile.

To determine the distance to the first neighbor, cells were fixed with PFA8% and stained with C-Laurdan. Wide field fluorescence images were acquired using an Olympus IX71 inverted epifluorescence microscope, with an UPlanSApo 10x objective (NA 0.30, Olympus). The images were obtained using a Qimaging EXI Aqua camera. The images were analyzed with an algorithm designed to binnarize the image and assign a label to each set of connected pixels. Every set of connected pixels represents a single cell in most cases and a cell cluster when the binnarization process could not distinguish among cells in contact or too close to each other. For every cell–cell cluster, the distance to all the other cell–cell clusters was calculated and to the first neighbor cell. Distances were normalized to the average cell radius.

Microtubule mechanics in PC3 cells was evaluated using confocal microscopy. Cells were treated with a primary antibody against tubulin and secondary antibody conjugated with Alexa-647. The effective Lp of microtubules in PC3 cells was determined using the methodology described by Gittes *et al.,*^37^ for cells treated or not with hemin. Briefly, microtubule xy positions were recovered using a filament tracking routine,^38^ a Fourier analysis was performed on the shape of the filaments and mode amplitudes were recorded. Finally, Lp* was obtained by fitting an inverse square law to the ensemble variance of these amplitudes as a function of the mode number.

#### Image processing for presentation

Confocal and wide field microscope images were processed for presentation using ImageJ software (NIH, Bethesda, MD, USA). Background of each channel was subtracted and in some cases a median filter (radius: 1 pixel) was applied only for presentation.

#### Immunoblotting

PC3 cells were lysed with CelLytic M Cell Lysis Reagent (Sigma-Aldrich, UK), incubated on ice for 20 min, centrifuged at 12 000 r.p.m. for 3 min and the supernatant was collected. Protein concentration was determined using the bicinchoninic acid (BCA) protein assay kit from (Sigma-Aldrich, UK). Samples were then resolved by SDS-PAGE, transferred to a nitrocellulose membrane (Invitrogen). Membranes were blocked for 1 h with 5% (w/v) non-fat milk in TBS-T buffer (0.1% Tween-20 in 10 mM Tris-HCl pH 7.4), and then incubated with specific primary antibodies overnight at 4 ^o^C: anti-HO-1 (1 : 1000), anti-*β*-actin (1 : 4000). The next day, the membranes were incubated with secondary antibodies for 1 h at room temperature. Protein bands were detected using ECL reagents (Amersham Ltd).

#### Cell tracking

Cells were seeded in 35 mm Petri dishes and cultured until confluence. The cells were then scraped with a 200 *μ*l micropipette tip, denuding a strip of the monolayer and placed onto a Zeiss Axio Observer Microscope (Zeiss, Germany) equipped with an incubation chamber (ibidi Heating System, Ibidi, Germany) set at 37 ^o^C. Phase contrast images were taken every 20 min during 48 h using a camera AxioCam HRm from Zeiss. The movies were processed using the cell tracking plug-in from Fiji (http://fiji.sc/Fiji) to extract cell trajectories and analyzed to obtain the MSD following equation 1, where x and y are the coordinates of the particle, t is time, and the brackets represent the trajectories ensamble average (see equation 1).


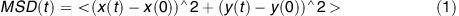


### Statistical analysis

Results are shown as mean±s.e.m of ‘*n*' separate independent experiments unless otherwise is stated. In cases were binning was required, the size of the bins was determined by Freedman–Diaconi's rule. Boxplots show mean, median and whiskers represent the 5–95% window. For the distance analysis to the first neighboring cell, distributions were compared with a two-sample Kolmogorov–Smirnov test. A kernel smoothing density estimate was applied to the data in order to obtain probability density estimate curves.

### RNA sequencing

The sequencing libraries for the RNA samples were prepared with the TruSeq Stranded Total RNA kit (Illumina Inc., San Diego, CA, USA), from 1 *μ*g total RNA. Following the manufacturer's instructions, the first step depleted rRNA from total RNA. After ribosomal depletion, the remaining RNA was purified, fragmented and primed for cDNA synthesis. Fragmented RNA was then reverse transcribed into first-strand cDNA using random primers. The next step removed the RNA template and synthesized a replacement strand, incorporating dUTP in place of dTTP to generate ds cDNA. AMPure XP beads were used to separate the ds cDNA from the second strand reaction mix resulting in blunt-ended cDNA. A single ‘A' nucleotide was then added to the 3' ends of the blunt fragments. Multiple indexing adapters, containing a single ‘T' nucleotide on the 3' end of the adapter, were ligated to the ends of the ds cDNA, preparing them for hybridization onto a flow cell. Libraries were purified and validated for appropriate size on a 2100 Bioanalyzer High Sensitivity DNA chip (Agilent Technologies, Inc., Santa Clara, CA, USA). The DNA library was quantitated using Kapa Biosystems qPCR kit (Wilmington, MA, USA), and then pooled together in an equimolar manner to a final concentration of 2 nM, following experimental design criteria. Each pool was denatured, diluted to 10pM, and clustered to individual lanes of a HiSeq Flow Cell using an Illumina cBot and the corresponding paired-end TruSeq V3 cluster kit. Pooled, clustered samples were then run on a HiSeq2500 sequencer according to the manufacturer's recommended protocol (Illumina Inc.).

## Figures and Tables

**Table 1:** List of HO-1-interacting proteins identified by MS/MS analysis

**Protein name**	**Gene symbol**	**Theroretical molecular mass (Da)**	**No. of peptides**	**Coverage %**
High mobility group protein HMG-I/HMG-Y isoform a	AHCTF1	11 669	2	29.2
Alpha-2-HS-glycoprotein	AHSG	39 193	5	25
Anexin 2	ANXA2	38 808	3	13.6
Cycle-like factor CLIF	ARNTL2	67 908	1	2
Aspartyl beta-hydroxylase	ASPH	85 959	1	3
Potassium-transporting ATPase alpha chain 1	ATP4A	114 045	1	2
Putative uncharacterized protein C5orf27	C5ORF27	10 849	1	14.58
Chromobox homolog 1	CBX1	21 519	2	11.2
Chromobox homolog 3	CBX3	20 997	2	15.2
Coiled-coil domain-containing protein 73	CCDC73	1079	1	2.78
Cardiomyopathy-associated protein 5	CMYA5	17 545	1	0.9
Damage-specific DNA-binding protein 1, 127kDa	DDB1	128 086	1	1.4
Translation elongation factor 1-alpha	EEF1A1	35 825	2	12
Eukaryotic translation elongation factor 2	EEF2	96 246	1	2.9
Translation initiation factor eIF3 p40 subunit	EIF3H	40 075	1	5
FtsJ3 protein	FTSJ3	84 015	1	2
Gelsolin	GSN	31 052	3	22.7
High mobility group AT-hook 1	HMGA1	34 635	1	5.1
Heme oxygenase 1	HO-1	32 798	5	25
Heat shock 27 kDa protein	HSPB1	22 427	3	31.4
Junctional sarcoplasmic reticulum protein 1	JSRP1	36 296	1	2
KH-type splicing regulatory protein	KHSRP	10 362	1	24.4
Lasp-1	LASP1	30 185	1	5
Matrin 3	MATR3	95 078	2	5
Multiple epidermal growth factor-like domains protein 10	MEGF10	122 121	1	2.11
Muskelin 1	MKLN1	84 713	11	17
Kinesin-like protein KIF23	MKLP-1	98 842	1	1.7
Interferon-induced Mx protein	MX1	75 929	1	2
Nucleosome assembly protein 1-like 1	NAP1L1	45 631	1	4.1
Nitric oxide-associated protein	NOA1	78 409	1	4.44
Nucleophosmin 1	NPM1	3109	2	12.7
5′-Nucleotidase, cytosolic II	NT5C2	65 384	1	2
Palmdelphin, isoform CRA_a	PALMD	53 404	1	2
Programmed cell death 5 short isoform	PDCD5	4472	1	32
Peroxiredoxin 2	PDX2	22 014	1	5
Purine-rich element binding protein A	PURA	35 003	1	8.2
Retinoic acid-induced 14	RAI14	110 617	1	1.5
Regulator of chromosome condensation	RCC1	44 485	1	3.7
Replication protein A 70 kDa DNA-binding subunit	RPA1	68 723	1	3.6
Ribosomal protein SA pseudogene 9	RPSA1	32 947	1	9
s100 calcium- binding protein A6	S100A6BP	1023	1	8
Splicing factor, arginine/serine-rich 3	SFRS3	19 546	1	11.8
Signal-induced proliferation-associated 1-like protein 1	SIPA1L1	201 102	1	1.25
K-Cl co-transporter KCC4	SLC12A7	120 327	1	0.6
Isoform 4 of Spastin	SPAST	54 385	1	7.23
Spermatogenesis-associated protein 7	SPATA7	8799	1	36.84
Sequestosome 1	SQSTM1	4837	1	4.5
RNA polymerase II transcription factor SIII subunit A3-like-2	TCEB3CL2	59 735	1	3.48
Testis-derived transcript	TES	49 789	1	3.1
Thyroid hormone receptor-associated protein 3	THRAP3	108 686	1	1.9
Mitochondrial import inner membrane translocase subunit	TIMM44	15 215	1	19.55
Tropomodulin 3	TMOD3	39 727	2	9.7
DNA topoisomerase 1	TOP1	66 890	2	5.3
Tripartite motif-containing 28	TRIM28	80 621	6	18
Zinc-finger CCCH-type, antiviral 1	ZC3HAV1	103 135	3	7.3
Zinc-finger protein 589	ZNF589	40 736	7	36
