# Supplementary material for: Heme oxygenase-1 in the forefront of a multi-molecular network that governs cell–cell contacts and filopodia-induced zippering in prostate cancer
Source: Cell Death Dis. 2016 Dec 29;7(12):e2570–. doi: 10.1038/cddis.2016.420 (PMC5261017; doi:10.1038/cddis.2016.420)
Supplement: Supplementary information [file cddis2016420x1.pdf]

Supplementary Figure 1

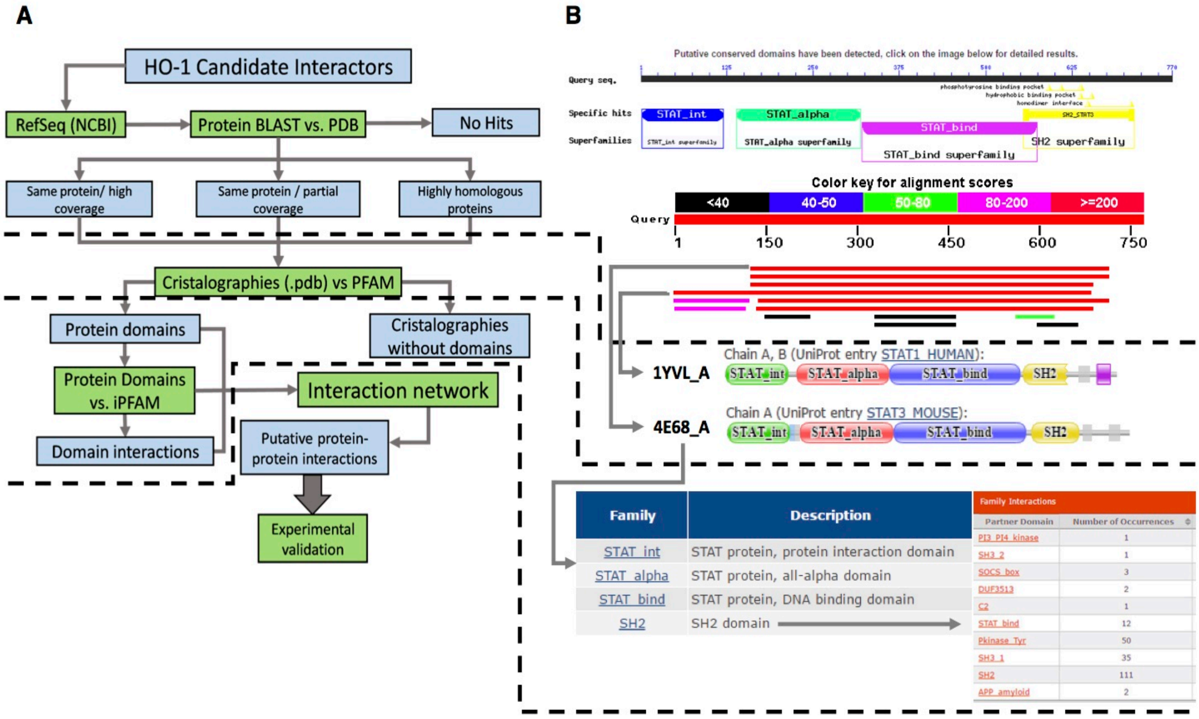

Supplementary Figure 1. Pipeline of protein domain analysis of HO-1 interacting proteins. (A) Schematic representation of working pipeline. (B) Reference amino acid sequences of protein domains of HO-1 interactors (Query seq, black bar) were aligned against the Protein Data Bank (PDB) obtaining a list of homologue crystallographies (red, pink, green and black lines) according to their coverage and score (top panel). The crystallographies with the highest coverage and score were selected and introduced into the protein–family database (Pfam) (middle panel). HO-1 candidate interactors protein domains were annotated (lower panel). Individual searches in the Interaction Protein Family (iPfam) database were performed for those domains and their interacting domains were also annotated. The figure shows STAT as an example of the analysis performed.

Supplementary Figure 2

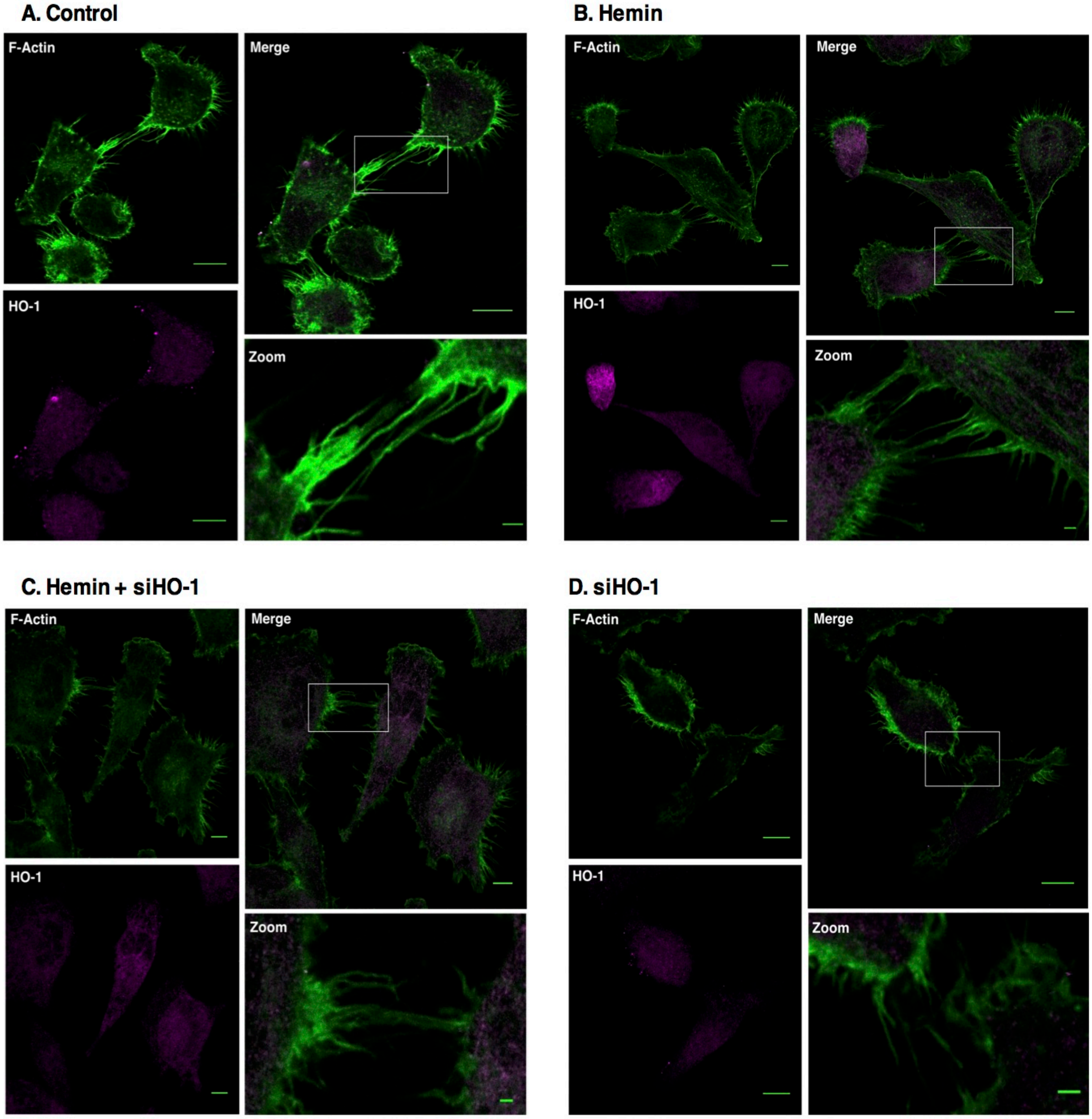

Supplementary Figure 2. HO-1 does not localize at cell filopodia in PCa cells. (A) Control PC3 cells or (B) Hemin treated PC3 cells (80μM, 24h), were transfected with a specific siRNA for HO-1 (siHO-1) (C & D). Cells were then fixed and stained with rhodamine-phalloidin for F-Actin filaments (green) and HO-1 (magenta) and imaged by confocal microscopy. One representative image for each group is shown. F-actin localization is observed in the cytoplasm but mainly at the cell membrane and at cell-cell adherens junctions revealing a network of filopodia from neighboring cells. HO-1 staining is observed at cell nuclei and cytoplasm with no staining at cell filopodia (scale bars: 10 μm). White open boxes represent the zoomed images (scale bars: 2 μm).

Supplementary Figure 3

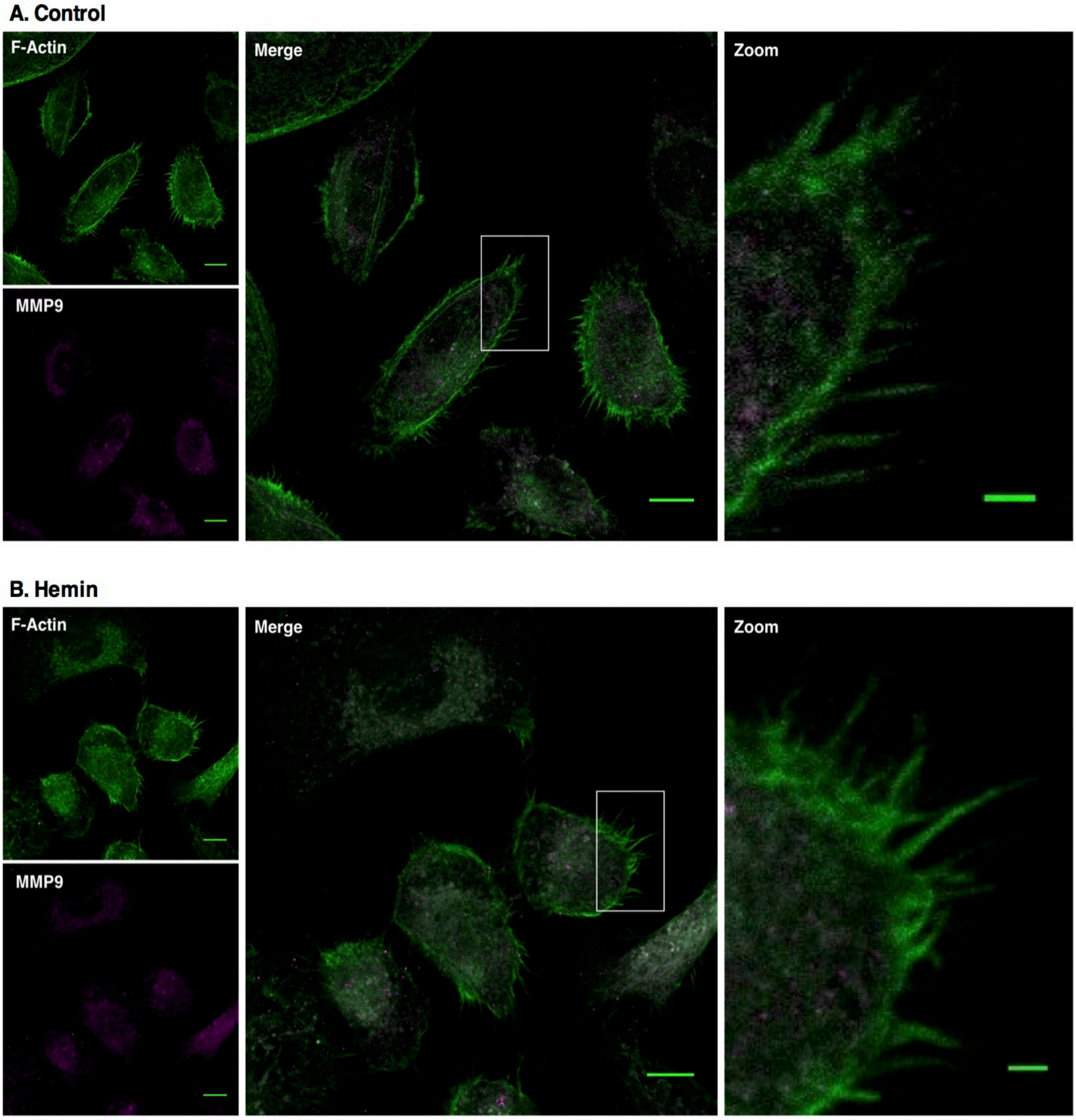

Supplementary Figure 3. MMP9 does not localize at cell filopodia in PCa cells. (A) Control PC3 cells or (B) Hemin treated PC3 cells (80 μM, 24 h) were fixed and stained with rhodamine-phalloidin for F-Actin filaments (red) and MMP9 (magenta) and imaged by confocal microscopy. One representative image for each group is shown. F-actin localization is observed in the cytoplasm but mainly at the cell membrane and filopodia. MMP9 staining is observed at cell cytoplasm with no staining at cell filopodia (scale bars: 10 μm). White open boxes represent the zoomed images (scale bars: 2 μm).

Supplementary Figure 4

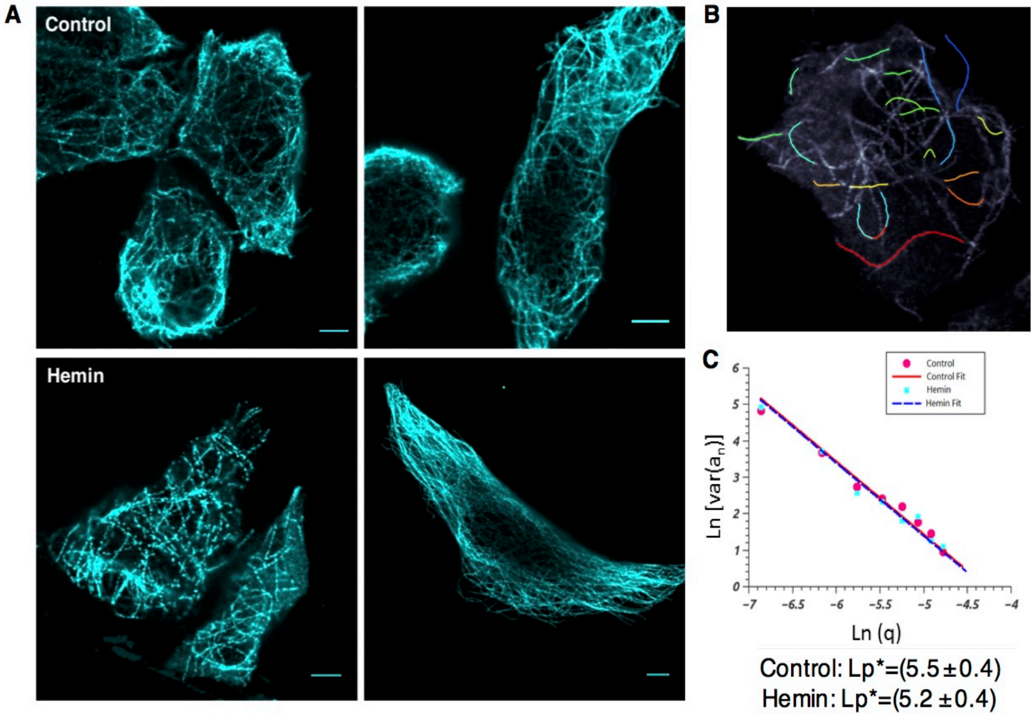

Supplementary Figure 4. Analysis of the persistence length of microtubules in PCa cells. (A) PC3 cells treated with hemin (80µM, 24h) or vehicle (control), fixed and stained with anti-tubulin primary antibody and secondary antibodies conjugated with Alexa-Fluor 647 (scale bars: 10 µm). (B) Representative image of the filament tracking on microtubules in a PC3 cell. (C) The Persistence Length of microtubules was measured in PC3 cells treated or not with hemin. Microtubule xy positions were recovered using a filament tracking routine previously described (Pallavicini et al. 2014). A Fourier analysis was performed and the ensemble variance of Fourier amplitudes obtained from fixed hemin treated (cyan squares) and control cells (pink circles) are shown. Both exhibit a thermal-like q-dependence: $(1/Lp^*)(1/q)^2$ (dotted lines). Lp*: effective persistence length, Ln: napierian logarithm, Var: variance q: wave number (see text for details).

Supplementary Figure 5

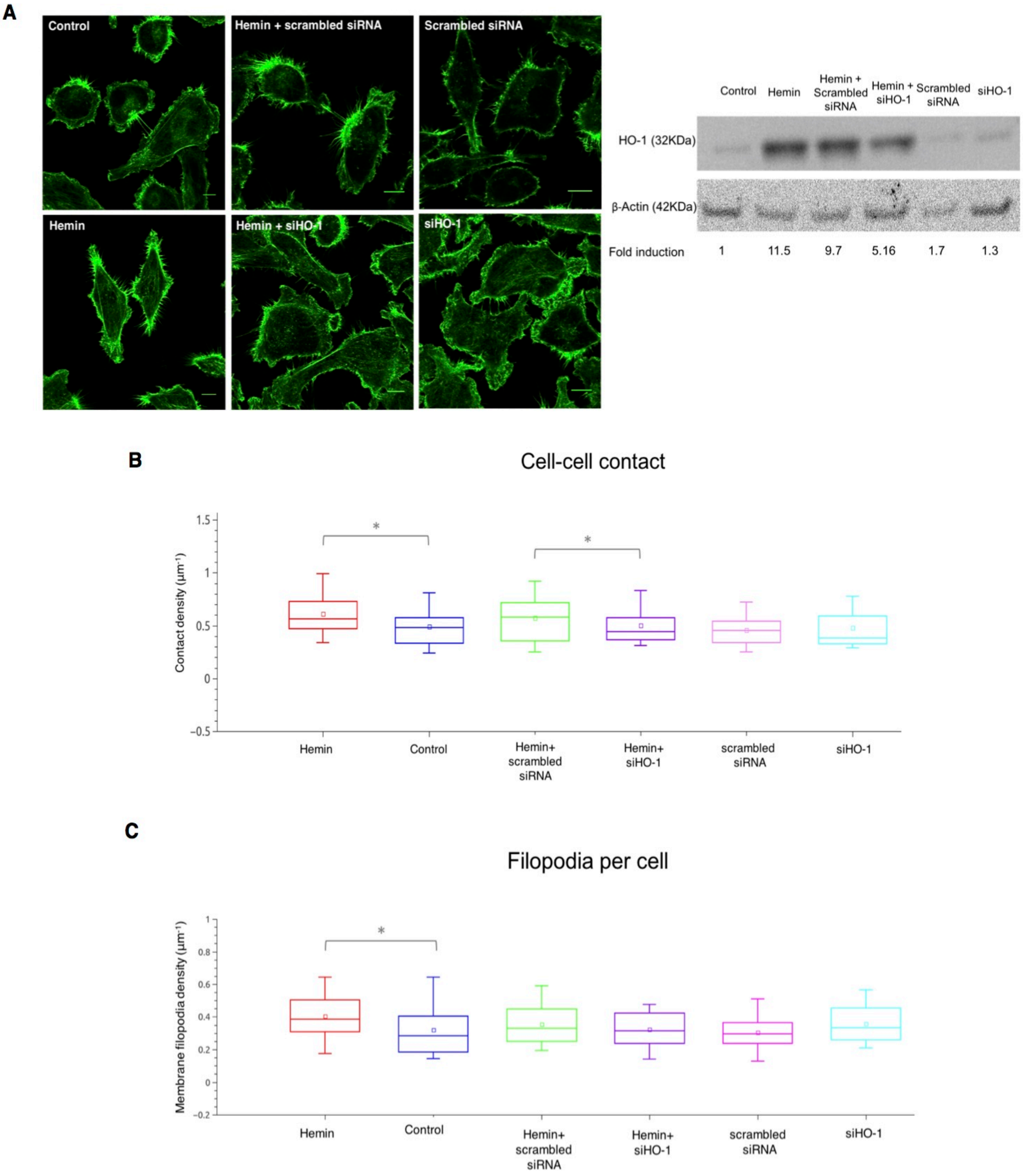

Supplementary Figure 5. Filopodia-like protrusions under HO-1 modulation using siHO-1 in C4-2B cells (a) C4-2B cells were treated with hemin (80μM, 24h) or vehicle (control) and transfected with a specific siRNA for HO-1 (siHO-1) or scrambled siRNA (control). Cells were fixed and stained with rhodamine- phalloidin and imaged by confocal microscopy. One representative image for each group is shown (left panel). Efficiency of siHO-1 was confirmed by western blot. β-Actin was used as loading control (right panel). (b) Boxplot comparing filopodia density on single cells for the different experimental conditions. The density was measured by scanning an intensity profile around the cell perimeter and evaluating the amount of filopodia per unit length. (N≥20 cells for each condition; *Significant difference, P<0.05). (c) Boxplot comparing the cell-cell contact density for the different experimental conditions. The regions, in which cell filopodia contacted two neighboring cells, were divided into segments where the distance between the cells remained constant. An intensity profile for each of these sectors was determined using a custom made algorithm to count contacts (Matlab) (N≥24 contact regions for each condition; *P<0.05).
